# Epidural cement leakage through pedicle violation after balloon kyphoplasty causing paraparesis in osteoporotic vertebral compression fractures - a report of two cases

**DOI:** 10.1186/1749-799X-5-54

**Published:** 2010-08-06

**Authors:** Si-Young Park, Hitesh N Modi, Seung-Woo Suh, Jae-Young Hong, Won Noh, Jae-Hyuk Yang

**Affiliations:** 1 Department of Spine Surgery, Orthopedic Department, Korea University Anam Hospital, Seoul, Korea; 2 Scoliosis Research Institute, Department of Orthopedics, Korea University Guro Hospital, Seoul, Korea

## Abstract

Kyphoplasty is advantageous over vertebroplasty in terms of better kyphosis correction and diminished risk of cement extravasations. Literature described cement leakage causing neurological injury mainly after vertebroplasty procedure; only a few case reports show cement leakage with kyphoplasty without neurological injury or proper cause of leakage. We present a report two cases of osteoporotic vertebral compression fracture treated with kyphoplasty and developed cement leakage causing significant neurological injury. In both cases CT scan was the diagnostic tool to identify cause of cement leakage. CT scan exhibited violation of medial pedicle wall causing cement leakage in the spinal canal. Both patients displayed clinical improvement after decompression surgery with or without instrumentation. Retrospectively looking at stored fluoroscopic images, we found that improper position of trocar in AP and lateral view simultaneously while taking entry caused pedicle wall violation. We suggest not to cross medial pedicle wall in AP image throughout the entire procedure and keeping the trocar in the center of pedicle in lateral image would be the most important precaution to prevent such complication. Our case reports adds the neurological complications with kyphoplasty procedure and suggested that along with other precautions described in the literature, entry with trocar along the entire procedure keeping the oval shape of pedicle in mind (under C-arm) will probably help to prevent such complications.

## Introduction

Osteoporotic vertebral compression fracture (OVCF) is the commonest complication of osteoporosis[1]. Over the past two decades, vertebroplasty was developed to stabilize OVCF without increasing morbidity and mortality associated with open surgery[2,3]. Diamond and colleagues. [4] noted that vertebroplasty for acute compression fracture was significantly better than nonoperative treatment in terms of pain relief, level of function, and hospital stay. However, complications related with vertebroplasty are not uncommon such as cement extravasation, pulmonary embolism, infection, epidural hematoma, systemic toxicity, and vertebral body fractures [5-17]. Among the commonest complications, cement extravasation has been estimated in 70% cases for vertebroplasty procedures [18,19]. Most of the time it is asymptomatic; however, disastrous complications causing paraparesis have been also reported in the literature [10,15,19].

Kyphoplasty, as a modification of vertebroplasty, has theoretical advantages such as focal kyphosis correction and diminished risk of cement extravasation due to lower cement injection pressures [9,11,20-22]. Backer et al. [23] reported that out of 100 balloon kyphoplasties, overall cement leakage rate was 31%. Most leakages were anterior and superior; only 2% were posterior and most leakages were below 3 mm. The biomechanical principle of increasing anterior column load with progressing kyphosis leading to subsequent vertebral compression fracture has established the basic rationale for kyphoplasty [24]. Probably that is the reason for increasing use of kyphoplasty procedures for OVCF now a day. Even though kyphoplasty has significantly lower rates of cement extravasations than vertebroplasty [21], cement leakage may occur more frequently than originally appreciated, and often associated with significant morbidity [25]. There are numerous descriptions of significant spinal cord or cauda equina injuries associated with vertebroplasty procedures; however, only a few reports have been described with kyphoplasty procedure [23]. Recently Patel et al. [26] reported 10 neurological complications with kyphoplasty procedure in a multicenter study, and suggested that physician should remain aware about such complications. The purpose of our case report was to present two cases of epidural cement leakage due to pedicle breakage causing significant neurological damage after the kyphoplasty procedure. We also aimed to address the reason caused pedicle rupture in both cases while taking entry into pedicle which can be prevented by simple care during procedure.

## Case report

### Case 1

**Figure 1:**
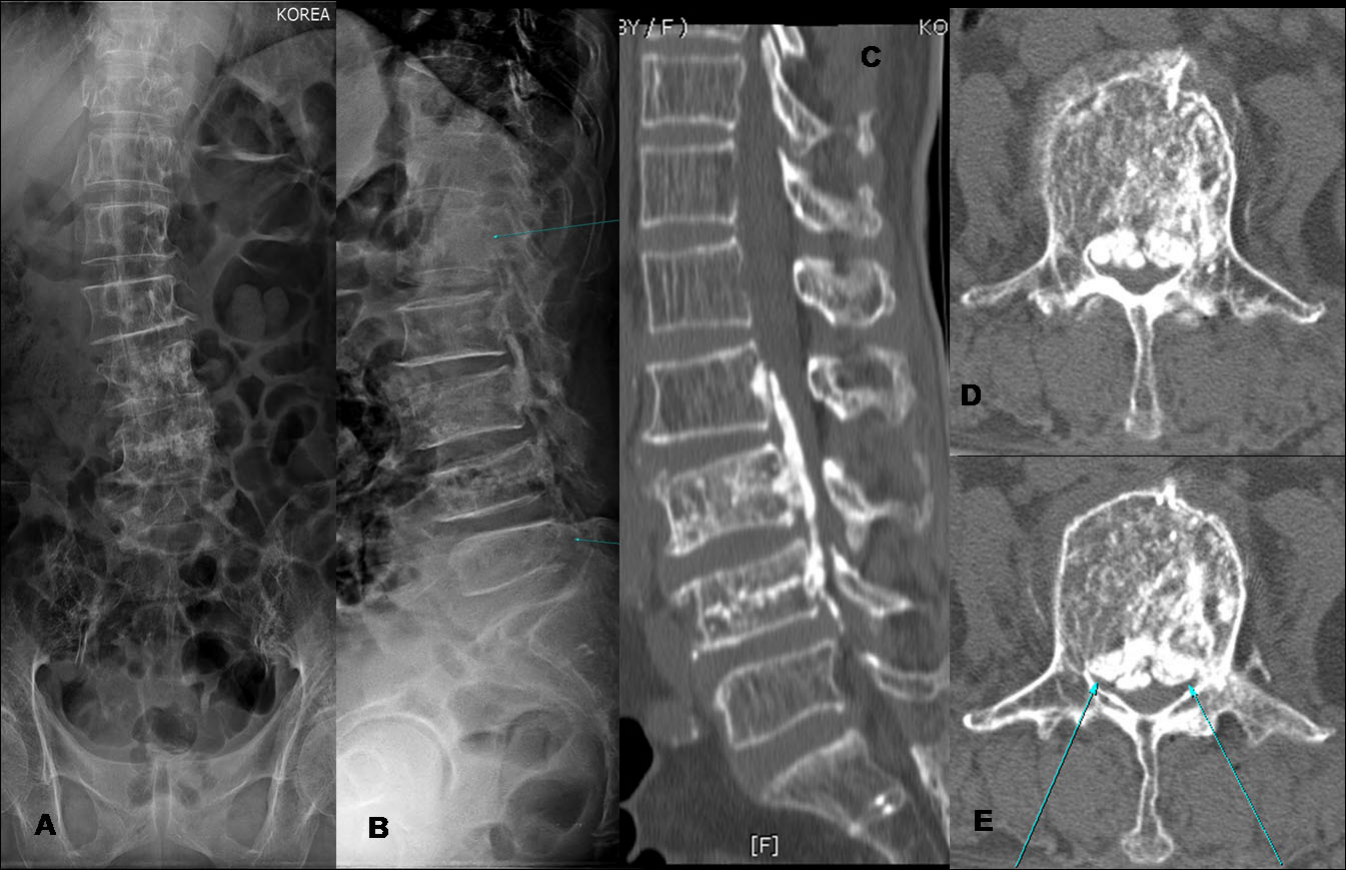
**(A-B) shows post kyphoplasty radiogram of lumbar spine which did not clarify any idea about cement leakage; figure (C-E) shows that cement leakage spread along the posterior longitudinal ligament at L2-L4 levels causing severe compression of canal, possibly through pedicle violation**.

An 88-year-old woman who was diagnosed with L3 and L4 OVCF received balloon kyphoplasty using PMMA in the neurosurgery department at local hospital. Two days after the kyphoplasty, she was sent to our department due to severe bilateral radicular pain in thigh and legs, with associated weakness and numbness in both lower extremities. She was unable to walk after the procedure which was in fact a new complaint after kyphoplasty. Referring physician informed about difficulty while taking entry into both the pedicles at L3 and L4. The procedure was performed with uniportal entry into pedicles. Examination revealed positive straight leg raising test in lower limbs, paresthesia and weakness in the both thigh and leg (muscle power: Grade 3) below L3, and restricted lumbar spine motion due to low back pain. Roentgenographic images (Figure 1A-B) showed the post procedure radiogram of lumbar spine which could not reveal further information about complication. Therefore she was investigated with CT scan (Figure 1C) which showed intracanal extension of cement from L2-L4 and axial image exhibit significant compression of cord at all three levels. Cement leakage was found from medial pedicle wall of L3 (Figure 1C-E) which was extended along the posterior longitudinal ligament at L2-L4 levels causing severe compression of canal.

**Figure 2:**
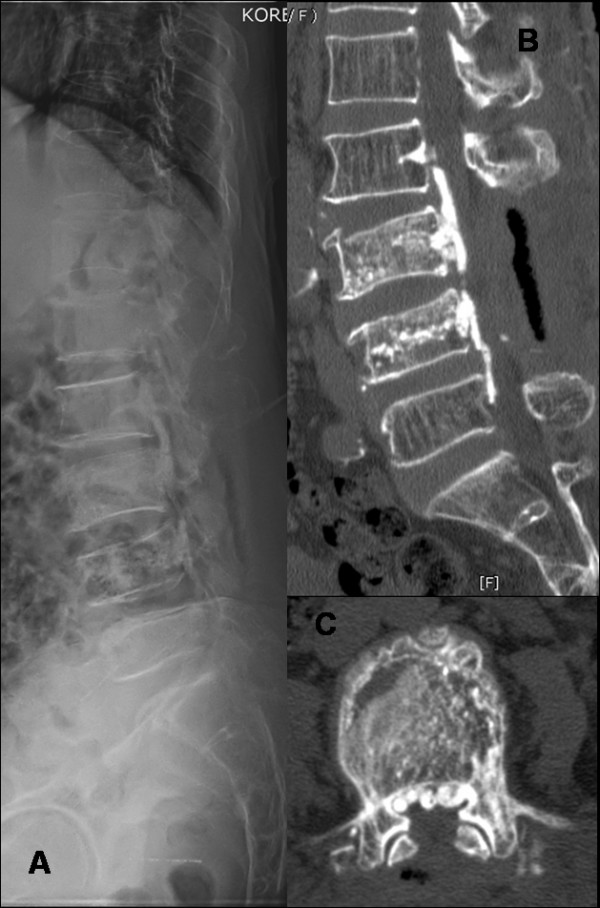
**(A-C) shows laminectomy of L3-and L4 and partial laminectomy of L2 performed to achieve decompression of cord without any instrumentation**. Complete removal of cement was not tried due to her health problem.

Surgical intervention was required to relieve the intractable leg and back pain as well as neurologic deficits. However, due to chronic renal failure and poor cardiac function of patient, one-stage posterior approach was performed without instrumentation: laminectomy of L3-and L4 and partial laminectomy of L2 was performed to achieve decompression of cord (Figure 2A-C). Complete removal of cement was not tried. During operation we could find the pedicle breakage from medial wall at L3 from where cement was removed. Nerve root decompression was performed at L2-L4 levels bilaterally and posterolateral fusion was achieved with local bone mixed with allograft. The radiating pain was immediately relieved after surgery. Five days after surgery, back pain was improved and motor weakness was recovered up to grade 4 bilaterally. The patient was discharged on the tenth postoperative day after suture removal. Six weeks after the operation, patient was able to walk slowly with the help of walker wearing TLSO brace. Muscle power in both the lower extremities was Grade 4 in thigh and legs. Still there was some paresthesia remaining in her left lower extremity compared to right on the latest follow-up.

### Case 2

**Figure 3:**
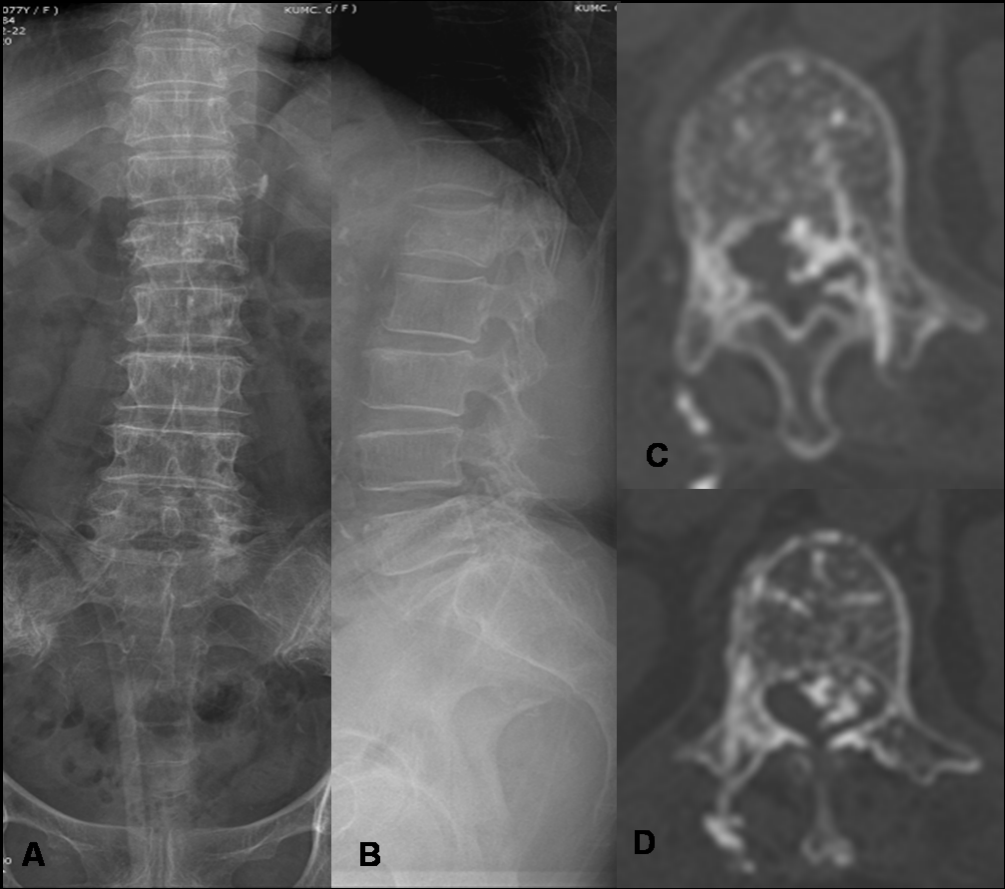
**(A-B) shows post procedure radiogram of lumbar spine which did not exhibit extravasation if cement into the spinal canal at L1 level; figure 3 (C-D) shows CT scan of lumbar spine that exhibited epidural extension of cement from medial wall violation of L1 pedicle on left side and causing significant compression of cord**.

**Figure 4:**
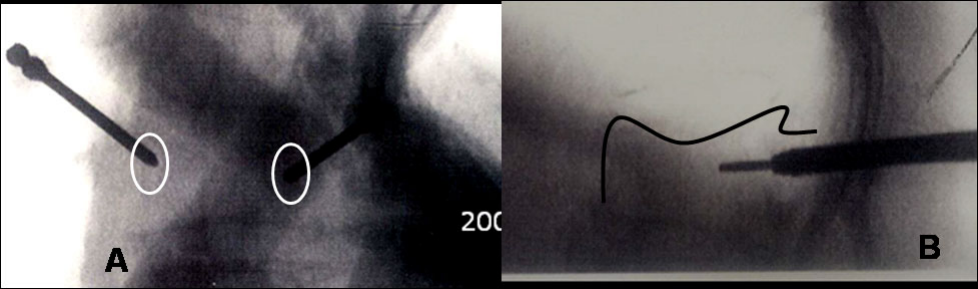
**(A-B) shows fluoroscopic stored images during the procedure which revealed that there was incorrect positioning of trocar cannula while taking the entry into the pedicle further confirming our suspicion of pedicle wall violation**.

A 77-year-old woman, with an L1 OVCF received balloon kyphoplasty using PMMA at our hospital. Kyphoplasty procedure was performed through biportal entry. Immediate after the procedure, her back pain was improved; however, she complained severe radicular pain in left thigh, with associated weakness and numbness in left lower extremity. The patient was unable bear weight on her left lower limb after the procedure; and her knee joint had giving way sensation on walking. Neurologic examination revealed a negative straight leg raising test in lower limbs; however, paraesthesia and weakness in the left thigh (muscle power: Grade 3) below L1 was significant. Right lower extremity did not reveal any positive neurological signs. Postprocedure roentgenographic images (Figure 3A-B) did not exhibit extravasation of cement into the spinal canal at L1 level; however, CT scan (Figure 3C-D) showed intracanal extension of cement from medial wall violation of L1 pedicle on left side and causing significant compression of cord. Retrospectively observing fluoroscopic images during the procedure revealed that there was an incorrect positioning of trocar while taking entry into the pedicle (Figure 4A-B); that further confirmed our suspicion having pedicle wall violation.

**Figure 5:**
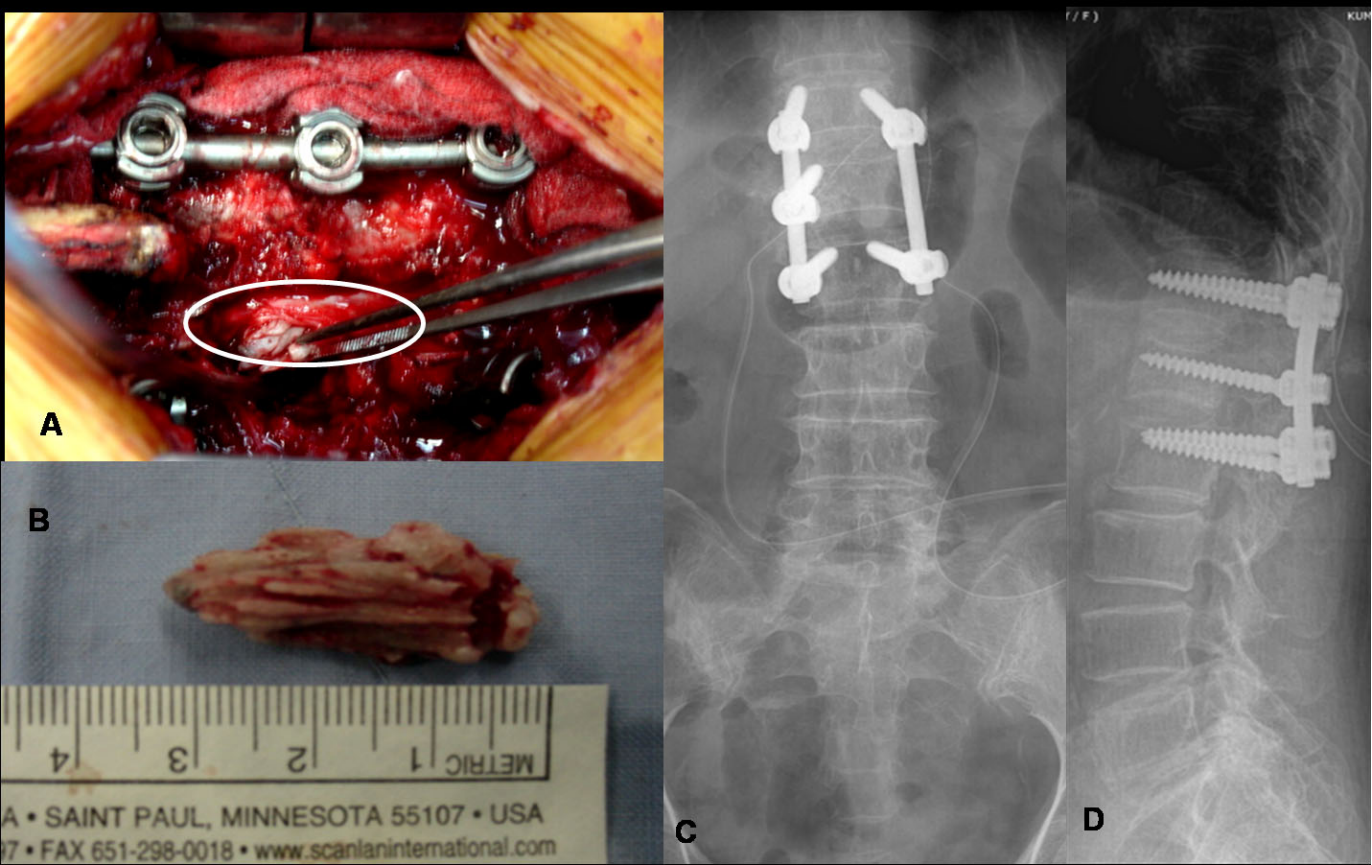
**A shows intraoperative image that showed epidural cement leakage from pedicle violation; figure 5B showed completely removed cement mass of around 3.3 cm size and figure 5C-D shows postoperative radiogram of lumbar spine after decompression and pedicle screw instrumentation followed by posterolateral fusion**.

Surgical intervention was performed using single-stage posterior approach: laminectomy of L1 was performed to achieve decompression of cord (Figure 5A-B) which was followed by pedicle screw fixation from T12-L2. Intraoperatively, we could observe epidural cement leakage from medial pedicle wall violation (Figure 5A). Complete removal of cement mass of around 3.3 cm size was done from left side (Figure 5B). Nerve root decompression was performed bilaterally, and pedicle screw instrumentation (Figure 5C-D) was done followed by posterolateral fusion using local bone mixed with allograft. The radiating leg pain was immediately relieved after surgery. Three days after the surgery, motor weakness recovered up to grade 4 on left side and paraesthesia was completely resolved. The patient was discharged on the tenth postoperative day after suture removal. Four weeks later patient was able to walk freely wearing TLSO brace. Muscle power recovered completely to Grade 5 at one month follow-up.

## Discussion

Epidural cement leakage has only been described for vertebroplasty so far [6], but it has devastating neurological effects in both vertebroplasty[10] and kyphoplasty[26] procedures. These complications require immediate surgical interventions with decompression, and if possible, removal of the cement causing compression [23]. In present report we have described two cases of epidural cement leakage following balloon kyphoplasy which caused significant neurological effects in both of them. Simple care could possibly have avoided this complication intraoperatively which has not been described in the literature.

Yeom et al. [27] described three different types of cement leakages after percutaneous vertebroplasty such as type B (via basivertebral vein), type S (via segmental vein) and type C (via cortical defect). They used CT scan to detect cement leakage; similarly, we have also used CT scan to detect cement leakage. However, they did not mention about leakage through pedicle wall. Unfortunately, C-arm fluoroscopy is the only way to monitor cement leakage during procedure. Furthermore, leak could be observed only in lateral not in AP view. Therefore, identifying the leakage under C-arm is really demanding in our opinion. Additionally it often difficult to judge the cement leakage on simple radiogram as it was seen in our case; and therefore, urgent CT scan should be recommended if we suspect any kind of leakage. Becker et al. [23] reported a case of cement leakage via pedicle wall perforation and mentioned oblique images would have been helpful to detect the leakage early. Nussbaum et al. [13] found that kyphoplasty may have an increased risk of pedicle fracture that can lead to spinal compression. They noted that at least five of the 20 spinal compression associated with kyphoplasty were caused by breakage of the pedicle during insertion of the cannula; and of the remaining 15 spinal compressions that developed, only two specified that a pedicle fracture was absent on postoperative imaging. Probably this could be one of the reasons that all previously described literatures with vertebroplasty have not mentioned this issue. As kyphoplasty is gaining popularity for OVCF, cement leakage due to pedicle fracture needs a special attention. In addition, risk to neurology cannot be ignored during kyphoplasty procedure although the literature mentioned low risk of cement leakage compared to vertebroplasty procedure [21,25].

With proper surgical technique the risk of cement leakage can be minimized. Greene et al. [28] popularized eggshell-technique, in which, after primary reduction with balloon a small amount of doughy cement is applied into the cavity, followed by re-inflation of the balloon. Their technique reported reduced risk of cement leakage. Anselmetti et al. [29] showed that high viscosity bone cement has low rate of cement leakage during vertebroplasty. However, in present both the cases; we used high viscosity bone cement. Hu et al. [30] suggested kyphoplasty via unipedicular approach to reduce cement leakage along the cannula tract. In our two cases, one patient (case 1) underwent kyphoplasty with uni-pedicular approach; which suggested us that there should be some technical problem which should be discussed. Pateder et al. [31] mentioned technical tips while taking entry into the pedicle and entering to the vertebral body. They suggested that, when drill/trocar is midway across the vertebral body in C-arm lateral view, AP image should be obtained; and in AP view, drill/trocar should be midway between the pedicle and spinous process at the same time. If it is closer to the spinous process, there is a chance of entering in to the spinal canal; similarly, if it is too close to the pedicle, it may be out laterally. However, we feel that to judge distance between spinous process and pedicle is difficult, and it cannot be helpful in patient who had previous laminectomy. It is rather easy to observe the oval pedicle shape in C-arm to judge proper positioning of trocar. In first case, history of difficulty while taking entry into the pedicle with trocar probably gave us information about pedicle violation. While in second case, observing the stored images in C-arm, we found when trocar was just posterior to the vertebral body in lateral view on left side, it was situated more medially to the pedicle in AP view; and that could be the reason for pedicle breakage even though the procedure was uneventful. As reported in literature [13], that kyphoplasty has higher chances of pedicle fracture; our patient might have pedicle fracture in subsequent procedure due medially located entry point. Therefore, we recommend observing the pedicle shape carefully while taking entry with trocar. Initially trocar should be located on the lateral margin of pedicle in AP view in the center and in middle of the pedicle in lateral view. And when trocar reached to posterior border of vertebral body in lateral view, again AP view is mandatory and trocar should not cross the middle of the oval pedicle shape. In this way when trocar reached up to anterior two-third of body (i.e. completion of entry) in lateral view, it should not cross the medial wall of pedicle in AP view.

In conclusion, due to high risk of pedicle fracture incidence during balloon kyphoplasty, risk of cement leakage via pedicle violation causing significant morbidity cannot be ignored. We have presented two cases with such complications during balloon kyphoplasty which suggested that along with other precautions described in the literature, entry with trocar along the entire procedure keeping the oval shape of pedicle in mind (under C-arm) will probably help to prevent such complications.

## Competing interests

The authors declare that they have no competing interests. Each author certifies that he has no commercial associations (e.g. consultancies, stock ownership, equity interests, patent/licensing arrangements, etc) that might pose a conflict of interest in connection with the submitted article.

## Authors' contributions

HNM has contributed in conception and design and acquisition of data, analysis and interpretation of data, drafting the manuscript and revising it critically, SYP has contributed in acquisition of data, revising the manuscript critically and given the final approval, SWS has contributed in conception and design of data, drafting the manuscript and given the final approval of manuscript, JHY has contributed in analysis of data and drafting the manuscript, WN has contributed in revising the manuscript, and JHY has contributed in interpretation of data and final approval of manuscript. All authors read and approved the final manuscript.

## Consent

Written informed consent was obtained from the patient for publication of this case report and accompanying images. A copy of the written consent is available for review by the Editor-in-Chief of this journal.
